# DEPRESCRIBING IN ASSISTED LIVING

**DOI:** 10.1093/geroni/igac059.993

**Published:** 2022-12-20

**Authors:** Barbara Zarowitz, Nicole Brandt

**Affiliations:** University of Maryland School of Pharmacy, Las Vegas, Nevada, United States; University of Maryland, Baltimore, Baltimore, Maryland, United States

## Abstract

Deprescribing is defined as the thoughtful process of “tapering, stopping, discontinuing, or withdrawing drugs, with the goal of managing polypharmacy and improving outcomes.” There are multiple clinical issues that deprescribing can address such as: antibiotic resistance caused by inappropriate and excessive use, the ongoing opioid epidemic, as well as over treatment particularly at the end of life. Networks have been established to address deprescribing across settings including assisted living nationally and internationally. Fourteen key informants from these networks were interviewed including different disciplines. From the interviews, six major themes across two domains were identified. The two domains included regional resources and knowledge gaps and the six themes included: (a) network structure, (b) public perception, (c) policy implications, (d) implementation, (e) challenges, and (f) recommendations. Overall, the importance of collaboration among interprofessional team members will be critical to the success of deprescribing as this clinical issue moves ahead.

